# Clay Ceramic Waste as Pozzolan Constituent in Cement for Structural Concrete

**DOI:** 10.3390/ma14112917

**Published:** 2021-05-28

**Authors:** Everton dos Santos Barreto, Karina Vaz Stafanato, Markssuel Teixeira Marvila, Afonso Rangel Garcez de Azevedo, Mujahid Ali, Ronald Matheus Lobo Pereira, Sérgio Neves Monteiro

**Affiliations:** 1 LAMAV-Advanced Materials Laboratory, UENF-State University of the Northern Rio de Janeiro, Av. Alberto Lamego, 2000, Campos dos Goytacazes 28013-602, Brazil; 202111220007@pq.uenf.br (E.d.S.B.); m.marvila@ucam-campos.br (M.T.M.); 201921220006@pq.uenf.br (S.N.M.); 2 Campus Campos dos Goytacazes, UCAM-Candido Mendes University, Rua Anita Peçanha, 100, Campos dos Goytacazes 28013-602, Brazil; 202111120024@pq.uenf.br; 3 LECIV-Civil Engineering Laboratory, UENF-State University of the Northern Rio de Janeiro, Av. Alberto Lamego, 2000, Campos dos Goytacazes 28013-602, Brazil; 4 Civil and Environmental Engineering Department, Universiti Teknologi PETRONAS, Perak 31750, Malaysia; mujahid_19001704@utp.edu.my; 5 Department of Materials Science, IME—Military Institute of Engineering, Square General Tibúrcio, 80, Rio de Janeiro 22290-270, Brazil; 202111220012@pq.uenf.br

**Keywords:** clay ceramic waste, pozzolan, concrete, properties, microstructure

## Abstract

Ceramic-based wastes generated from different industrial activities have increasingly been reused as construction material incorporated into concrete. In general, these wastes just replace common concrete aggregates such as sand and gravel. In the present work, waste from clay brick industries composted of kaolinite minerals were for the first time evaluated for their potential to be reused as the pozzolan constituent of a cement for structural concrete. Initial standard testes revealed that the clay ceramic waste (CCW) displays high pozzolanicity. Concrete was then produced with 10 and 20 wt.% of CCW mixed with ordinary Portland cement (OPC) as its pozzolan constituent. Compression strength of these concretes and of pure OPC as a control sample were determined in standard tests after 14 and 28 days of curing. In addition, the corresponding density, water absorption, capillarity and percentage of voids were measured together with the evaluation of microstructural indices by scanning electron microscopy. The initial tests confirmed that the CCW is indeed an effective pozzolanic potential due to a chemical effect by reacting with CH to generate C–S–H. Moreover, the technological results proved that CCW might effectively replace the pozzolan cement constituent for structural concrete.

## 1. Introduction

The study of materials with pozzolanic potential is a major focus in the application of cementitious materials, within the materials of science [1,2]. This is because pozzolanic materials might replace clinker or cement, leading to a great environmental and economic gain [3,4]. Ordinary Portland cement (OPC) is known to be the second most consumed material in the world and has a large amount of gases emitted in the greenhouse effect [5,6]. That is why it is important to look for alternative materials to replace the OPC.

Pozzolans, naturally found or as waste, are defined as materials that do not have agglomerating power on their own but finely ground in the presence of cement and water and have the potential to undergo hydration reaction for consolidating as a solid material [7]. For this purpose, in general, they have a siliceous or silico-aluminous chemical composition. Examples of industrial waste pozzolans are: rice husk ash [8,9] and sugar cane bagasse [10,11], and natural materials such as calcined clay [12] and volcanic ash [13,14]. In this context, it is worth noting that the use of pozzolanic materials together with cement optimizes the material’s properties, in fact when pozzolanic materials are alone, they do not have binding properties. Therefore, it is common for waste usually discarded without applications to be incorporated into construction materials such as ceramic bricks or cement soil blocks, when used in conjunction with cement. In addition, the application of wastes with pozzolanic behavior adds commercial value to a material that is initially considered as garbage for disposal.

The pozzolanic reaction that occurs in the materials with this behavior are considerably complex [15], mainly due to the variability of physical–chemical characteristics that these materials present and the interference of parameters such as chemical, mineralogical, fineness, granulometry and specific surface area [16]. There is an already established understanding of the functioning of this type of reaction for the case of OPC. In general, the steps of cement hydration that make it possible to obtain resistant compounds are based on the reaction [17,18]:

OPC + Water → C–S–H + CH + Heat(1)

C–S–H is hydrated calcium silicates and CH is portlandite.

The release of heat indicates that Equation (1) is exothermic. C-S-H is the main component responsible for the strength of the concrete. CH (portlandite) has a secondary role in the strength of concrete, when compared to the C–S–H, also presenting contributions with regard to the durability of the material [19]. With the use of a pozzolanic material (S), the cement hydration reaction takes an additional step:

CH + S → C–S–H(2)

It should be noticed that with the use of pozzolanic materials, or simply pozzolans, there is a readjustment in the amounts of C–S–H obtained in the final product, which will naturally contribute in some way to the strength of the concrete [20,21]. This allows the use of smaller quantities of OPC, which considerably assists the sustainable development in the civil construction sector. In addition, the application of pozzolans reduces the heat of hydration, improving shrinkage and reducing the cracking susceptibility of the material. The whole world knows about these properties and applies pozzolans on cement, generating the so-called composite cement, standardized by Brazilian and international standards [22,23,24].

In Campos dos Goytacazes, in the north of the state of Rio de Janeiro, Brazil, the red ceramic manufacturing process, which involves manufacturing, transporting and storage, generates approximately 19 thousand tons of waste per month [25]. However, the proper management of these wastes is still a procedure that is not widespread in the local ceramic industry. Studies show that there is a great potential of adding value to the ceramic waste [6]. This is one of the objectives of this work, which aims to propose a methodology to assess the pozzolanic potential of ceramic waste, in order to use the material instead of clinker or cement for structural purposes.

Some important works have already been carried out with this type of waste in construction materials [26,27,28,29,30,31,32,33]. Azevedo et al. [26] proposed using the ceramic waste as a geopolymeric activator, obtaining a construction material with interesting technological properties and which presents application as tiles. However, due to the use of alkaline activators, which are materials with considerable price, the application of the waste in geopolymers is limited. Rashid et al. [27], Ogawa et al. [28] and Huseien et al. [29] investigated the use of ceramic waste in concrete to replace coarse aggregates, carrying out a studies of mechanical and rheological properties. These studies obtained promising results, but the waste was used as a substitute for coarse aggregate, which is an environmentally problematic material in the production of concrete. Keshavarz and Mostofinejad [30] conducted a similar study using porcelain ceramic waste. Bommisetty et al. [31], Zareei et al. [32] and Medina et al. [33] proposed the incorporation of ceramic waste from different sources, such as tiles, blocks and sanitary wares. The authors performed the replacement of aggregates by ceramic waste, obtaining excellent results of mechanical strength. A major limitation in these works was the use of ceramic waste as a substitute for aggregates and not for OPC, despite the great potential that the material presents. This is the proposal of the present work. This is one of the research gaps that are being studied in this work: the application of ceramic waste as a supplementary material to cement, or as a pozzolanic mineral additive in OPC-based materials.

Regarding the specific application of ceramic waste as a pozzolan material to act as OPC for structural concrete, Table 1 presents main works on this subject. A wide variation in the type of waste and related composition are highlighted in these works [34,35,36,37,38,39,40,41,42].

Kannan et al. [34] studied a waste from polishing enamel ceramic composed of 69.4% SiO_2_ and 18.2% Al_2_O_3_. Irassar et al. [35] investigated an Argentinean industrial waste mostly of quartz, 51.3% SiO_2_, 20% Al_2_O_3_ and 11.5% CaO and feldspar, anorthite and hematite. Pokorný et al. [36] evaluated the pozzolanic potential of Spanish industrial ceramic block waste, which was characterized by an elevate content of gypsite, 37% SiO_2_, 14.3% Al_2_O_3_, 15.2% CaO and 17.9% SO_3_, in addition to microcline, albite and muscovite. With similar work as in [35], Vejmelková et al. [37] used thermal insulation block waste. In two complementary works, Cheng et al. [38] and Chen et al. [39] applied the same ceramic waste with quartz and mullite, 60.02% SiO_2_, 16.04% Al_2_O_3_, 4.08% MgO and 3.15% Na_2_O. Pacheco-Torgal and Jalali [40] considered a ceramic waste composed of quartz, hematite, calcite and others mineral as pozzolan for cement production. In the words of Reiterman et al. [41] and Wang (2009) [42] the respective wastes were not characterized.

Based on these aforementioned ceramic wastes, kaolinite clayey waste has so far been considered as pozzolanic material. Another relevant point highlighted in Table 1 is the relatively high amount incorporated, up to 60% [37]. However, Kannan et al. [34] indicated that amounts lower than 30% are technical viable. Irassar et al. [35] concluded that the incorporation of 16% of the ceramic waste is viable. Similar conclusions were reported in the other research works listed in Table 1. This is apparently a consequence of the chemical composition of a hardenable cement with around 55% of C–S–H and 8–10% of ettringite and other compounds such a calcium mono sulfate aluminate 5% and secondary aluminate and ferrite phases 8%. This composition results in 22–24% of CH in the finally hardened concrete [43,44]. As material by its own definition, in a pozzolanic material only the CH is reactive up to these values. Higher amounts causes a loss in the concrete strength. Therefore, in the present work it was decided a kaolinite clay ceramic waste substitution of only 20 wt.% for the OPC would be used.

Another important point regarding the results present in Table 1 is that the pozzolanic potential of the investigated ceramic waste [36,37,38,39,40,41,42] was indirectly related to the decrease in strength of the hardened concrete. As exceptions, in [34,35] the authors applied the Frattini test to evaluated the pozzolanic potential. Actually, this is not exactly a direct measurement of such potential indeed, the Frattini test evaluated the pH resulting from a water solution containing 20 g of cement paste, used to fabricate the concrete, after 28 days of cure. All pH modifications are attributed to pozzolanic reactions and correlated to its potential. However, it is known that other factors not necessarily related to these reactions may modify the pH by forming a late ettringite or precipitation of calcium mono sulfate aluminite [43,44]. As such, none of these works listed in Table 1 has directly measured the pozzolanic activity of their respective ceramic waste. Therefore, in addition to the clay ceramic waste, this works presents another innovative aspect associated with the reliable Luxán method of evaluating the pozzolanic potential.

In this context, it is highlighted that the main objective of this work is to evaluate the application of ceramic waste as a substitute for Portland cement in concrete, evaluating the pozzolanic potential of the material through results of compressive strength, microscopy and Luxán method. The main research gap is the characterization of the ceramic residue using methods that evaluate the pozzolanic activity of the material by a coherent procedure, such as the Luxán method, different from other authors, such as those highlighted in Table 1, which evaluated the residue by the Frattini test procedure.

## 2. Materials and Methods

**Figure 1:**
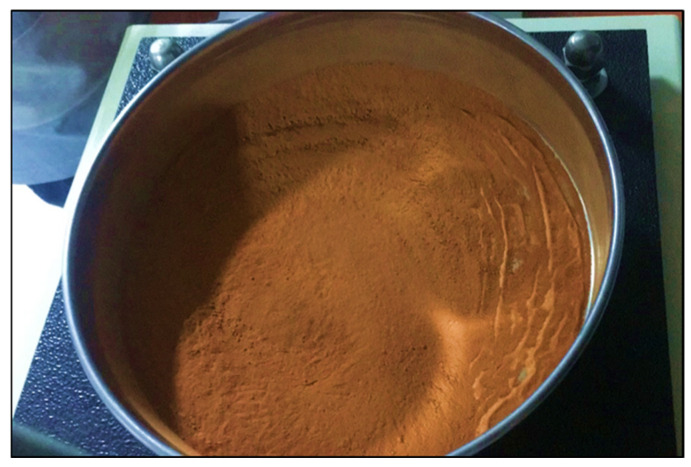
Clay ceramic waste (CCW).

To carry out this study, clay ceramic waste (CCW) was used, see in Figure 1. This waste was generated from ceramic blocks fabricated in the Baixada Campista ceramic industry, located in the city of Campos dos Goytacazes, Brazil. The material was crushed in a ball mill to acquire granulometry similar to that of cement and sieved through the #100 sieve, in order to standardize its granulometry.

**Figure 2:**
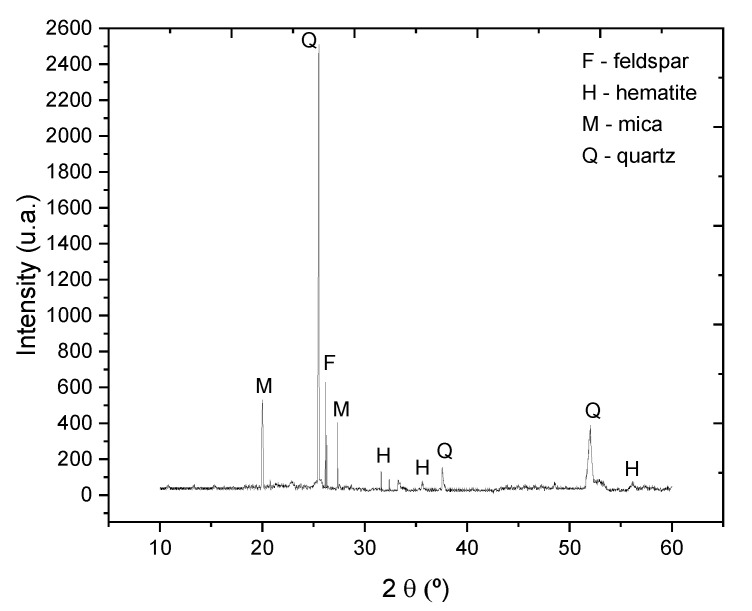
X-ray Diffraction (XRD) pattern of waste CCW sample.

The waste (CCW) was characterized chemically by X-ray Fluorescence (XRF), as seen in Table 2, and mineralogically (XRD), shown in Figure 2.

Can be observed in Table 2 that waste has great amounts of SiO_2_ and Al_2_O_3_, besides the presence of other minor oxides such as Fe_2_O_3_, Na_2_O, K_2_O, P_2_O_5_, TiO_2_ and CaO. In Figure 2 were can be peaks reveal the presence of mica (M), feldspar (F), quartz (Q) and hematite (H). The absence of kaolinite can be explained by the fact that the material was fired at 600 °C, occurring the transformation of kaolinite into metakaolinite (amorphous phase).

The CCW had its pozzolanic activity evaluated through the Luxán method of the conductivity measurement [45]. In this method the conductivity measurement of the initial and final material is carried out in an interval of 120 s in a calcium hydroxide solution causing a reaction of the material with the free ions of the solution [45,46]. The test is done with the powdered waste, in the same granulometry in which it will be applied to the concrete, simulating the occurrence of the pozzolanic reaction in the material. The final result of the Luxán method is the difference between the final and initial conductivity, between the 120 s interval, which classifies the material according to its pozzolanicity.

Three concrete compositions were produced using CP III-40 RS cement, a commercial Brazilian OPC (Votorantim Cimentos, Cantagalo-RJ, Brazil). Owing to the reduced presence of pozzolans, favoring the evaluation of the activity, coarse aggregate of the type gravel 1 and fine aggregate of the type natural sand washed from the Paraíba do Sul river with grains of 0.01–2.4 mm in diameter were also included into the concrete. The concrete compositions were selected as 0%, 10% and 20 wt.% of CCW in substitution for OPC, as indicated in Table 3. With the concrete in the fresh state, the cone trunk sag test was performed [47]. The determination of the consistency by the dejection of the cone trunk was carried out by filling of the standard cone trunk in three layers of equal height, and in each layer 25 blows were made with a standard stem and the abatement value was the height between the densified concrete after its spreading and the height of the standard cone [47]. The specimens were molded as cylindrical molds 10 cm × 20 cm, through filling and densification in three layers [48]. After molding, the specimens were cured by immersion in a tank with calcium hydroxide, and tests were performed at 14 and 28 days of curing. In each test were used 3 samples, because this is the number of specimens that is stipulated by the Brazilian technical standard for quality control of concretes and determination of statistical deviations [48].

For the execution of the technological tests, the specimens were removed from the submerged cure 30 min before, with a cloth the excess surface water was removed, to allow the execution of the tests. After, compressive strength tests were carried out after 14 and 28 days of curing, using a Solotest hydraulic press with a speed of 50 N/s, for 3 specimens in each mixture [49]. Neoprene discs wrapped in metal plates were used to smooth the surfaces of the specimens. Thus, it was also possible to obtain the index of pozzolanic activity (IPA), using the procedures of the Brazilian standard that require that a minimum percentage index of 75% be used for the calculation to establish whether the wastes used have pozzolanic capacity or not [49].

Mass density tests in the hardened state, capillary water absorption tests and immersion and boiling water absorption tests were also performed, in all of these tests 3 specimens were made for each mixture [50]. The purpose of these tests was to obtain concrete densification parameters and to check if there was any filling effect caused by the waste, a characteristic also attributed to pozzolanic materials. The density of the mass in the hardened state was determined by dividing the mass by the volume of each specimen, the mass were obtained on a scale and the volume measuring the respective dimensions (width and height) of the specimens [51]. The determination of water absorption by capillarity was performed after sanding the base of the specimens, followed by the determination of their initial mass. Right after the specimens were positioned vertically in contact with a 5 mm constant water slide, and after 3, 6, 24, 48 and 72 h new masses were determined, making it possible to determine the water absorption [52]. The absorption of water by immersion in boiling water was determined by comparing the mass of the dry specimen, obtained after 72 h in an oven at 110 °C (saturated mass), and the mass obtained after immersion of the specimens in boiling water for a period of 5 h (saturated mass). The absorption of water by immersion in boiling water was obtained by the difference between the mass saturated with the dry mass, divided by the dry mass [50].

Finally, a microstructural analysis was performed using scanning electron microscopy (SEM) in a Jeol microscope equipment model JSM 6460 LV (JOEL, Tokyo, Japan) for complementary characterization of the material. For microstructural analysis the reference mixture (without any addition) and the optimal mixture were selected, which had the best technological properties, according to the results found.

## 3. Results and Discussion

Table 4 presents the results of the pozzolanic activity of the investigated CCW. It should be noted that the values of pozzolanic activity obtained by the Luxán method were classified as follows: (i) if the value of the resistivity variation was less than 0.4 mS/cm the material was not pozzolanic; (ii) if the value was greater than 1.2 mS/cm, the material was highly pozzolanic; (iii) if the variation in resistivity was between 0.4 and 1.2 mS/cm, the material was medium pozzolanic. As can be seen in Table 3, the values obtained were of a highly pozzolanic material. These results were related to the chemical and mineralogical composition of the CCW. As reported by Azevedo et al. [26] that used the same waste for applications in geopolymers, i.e., CCW presents similar chemical composition. Adding the percentage of these oxides (SiO_2_ + Al_2_O_3_ + Fe_2_O_3_), a 92.80% ratio was obtained. This was above the limit of 70% established to classify the material as class N pozzolan, which is the most noble class of pozzolans, calcined clays and materials of volcanic origin [53]. To understand the mineralogical composition, it is necessary to know the burning temperature of the material. This was reported by Azevedo et al. [26], to be around 600–700 °C in the region where the material was studied. In this same research, the authors point out that the mineralogical composition of the material was characterized by the presence of metakaolin and amorphous regions. It is known that the degree of amorphism was directly related to pozzolanic activity, since the pozzolanic reaction was favored by this type of material. Thus, it was found that the investigated CCW was suitable for use as a pozzolan in concrete.

**Figure 3:**
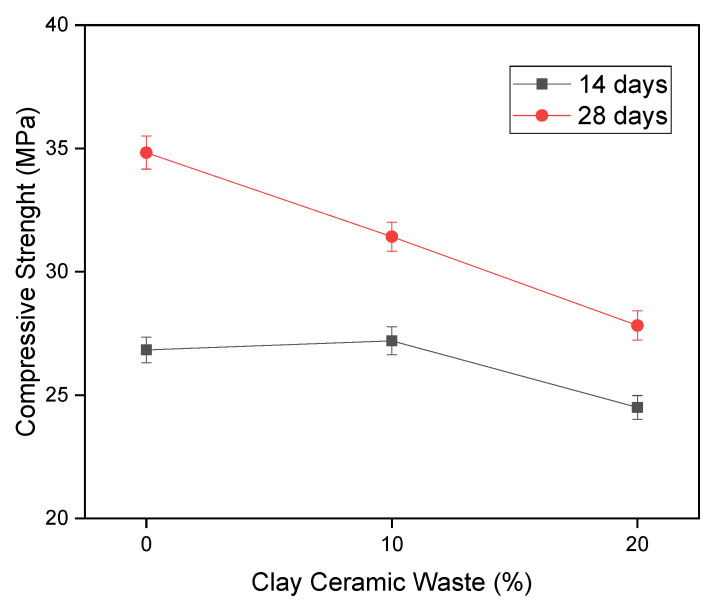
Compressive strength.

Figure 3 shows the results of compressive strength of the concretes evaluated in this research, by their strength values at 14 and 28 days. It appears that at 14 days all compositions revealed strength values above 24 MPa, and the composition of 10% displayed statistical equivalence with the percentage of 0% both at 14 and 28 days. The increase in composition 0% to 10% was only 0.37 MPa to 14 curing days, being within the margin of error. By contrast, in the case of the composition with 20% it is observed a drop in strength to values that are still tolerable. With the results obtained by the compression test, it is possible to discuss two items relevant to the application of ceramic waste as pozzolans in concrete composites. These are the index of pozzolanic activity (IPA) and the characteristic strength to compression.

In relation to the pozzolanic activity index, according to the Brazilian norms [53], 75% of the value obtained by the reference composition should be used to prove the pozzolanic activity of the waste under study. In the case of compression strength at 14 days, 26.83 MPa was obtained for the 0% composition. Evaluating 75% of this value, the limit of 20.12 MPa was obtained. As such any composition evaluated that presents a strength greater than 20.12 MPa should be considered pozzolanic. The concrete C10% showed strength at 14 days of 27.2 MPa, while C20% showed 24.50 MPa, indicating that both have pozzolanic characteristics at 14 days of curing. At 28 days, the reference composition obtained a strength of 34.83 MPa, with the limiting value of 75% being 26.12 MPa. Observing that the compositions with 10 and 20% of the CCW showed strength at 28 days of 31.42 and 27.82 MPa, respectively. Therefore, it is observed that the CCW acted as pozzolanic material, once the 75% limit was met by the standard established compression strength.

Another important characteristic observed in Figure 3 is that the compressive strength values at 14 days were statistically equivalent for the composition of C0% and C10%, as observed by the error bar. At 28 days, however, there was a statistical difference and the C0% composition was more resistant than the C10% composition. This happens because the evaluated residue increased the kinetics of the hydration reaction, due to its pozzolanic properties.

Regarding the characteristic compression strength, it is noteworthy that this is the main parameter used in structural engineering projects. This parameter was obtained from the experimental results of compressive strength. As defined by the Brazilian standard, this characteristic *fck* strength of concrete is the value that presents a 95% probability that individual strength values of test specimens are higher, that is, only 5% of the values are less than or equal [54]. Assuming the hypothesis of normal statistical distribution of strengths, the previous definition leads to the adoption of the 5% quantile value for the characteristic compression strength value (*fck*). This rule is applied through the Gauss curve, using the following equation:

(3) fck=fcm−1.645×S

*fcm* represents the average strength obtained in the compression test at 28 days and the *S* represents the standard deviation related to that test.

After applying the values obtained for strength to 28 days for the compression test in the CCW compositions of 0, 10 and 20%, 28.98, 27.06 and 25.86 MPa were obtained, respectively. According to Brazilian standards, concrete is considered structural when it has more than 20 MPa of *fck*, which leads to the conclusion that all compositions can be used as structural concrete [55]. Additionally, in more aggressive environments, such as urban pollution, typical of big cities, the concrete must present at least 25 MPa of *fck* [56], which in the present case was also verified for all studied compositions. Thus, it appears that, even with a drop in compressive strength, in the case of 20% CCW, the concrete had pozzolanic characteristics because even with the replacement of OPC it was possible to obtain satisfactory mechanical parameters for real applications of the material in structural projects.

**Figure 4:**
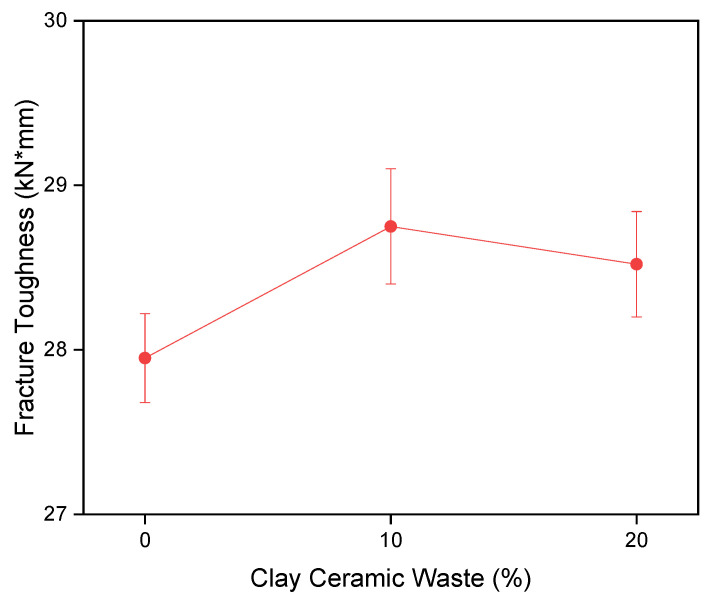
Fracture toughness.

Figure 4 shows the results of fracture toughness obtained by an estimate of the mechanical behavior of concretes, through the compression results highlighted in Figure 3. It is observed that the composition C0%, C10% and C20% present fracture toughness statistically equivalent, indicating that the use of ceramic residue did not alter the mechanical properties of fractures of the studied concretes. This behavior was positive because it indicates that the ceramic residue was a substitute equivalent to Portland cement, when taking into account the fracture toughness information highlighted in Figure 4.

**Figure 5:**
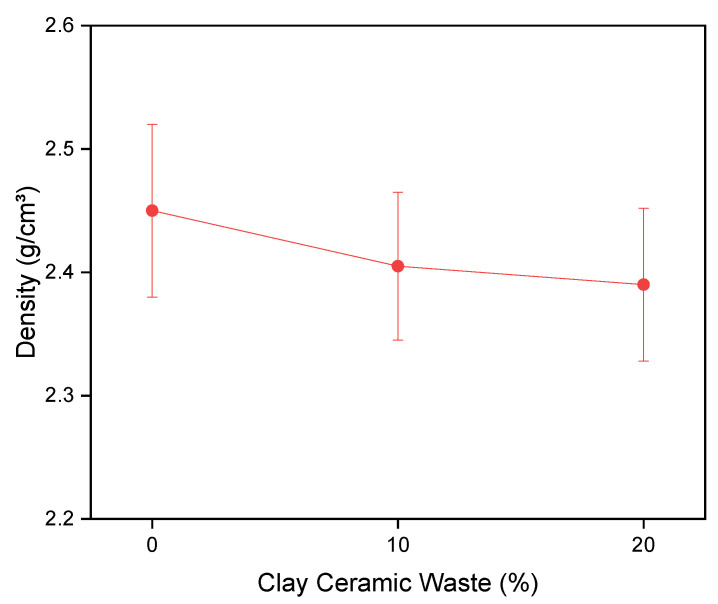
Results of density.

**Figure 6:**
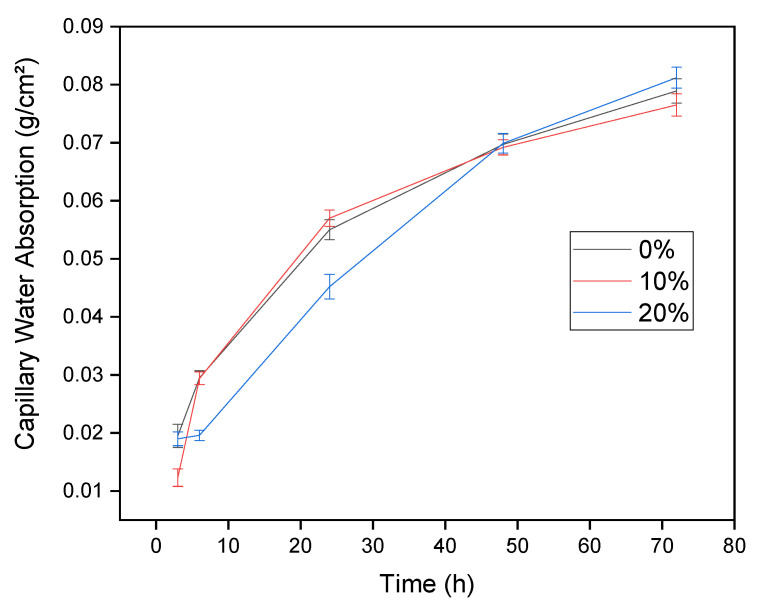
Capillary water absorption.

**Figure 7:**
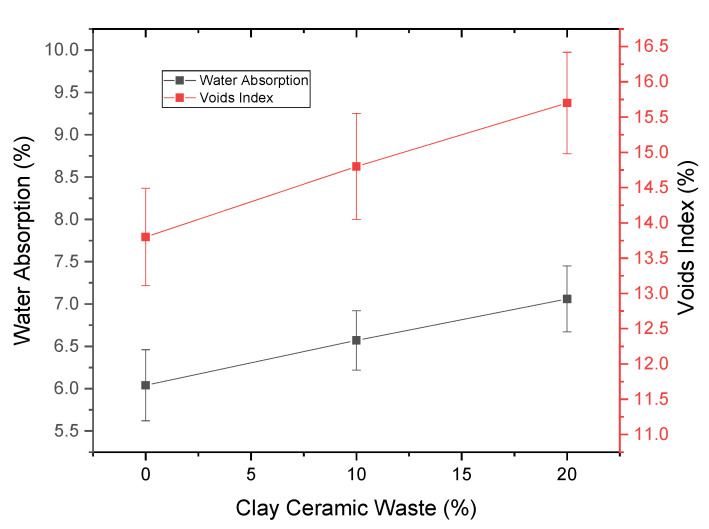
Water absorption and voids index.

Figure 5 shows the density while Figure 6 shows the capillarity results and Figure 7 shows the water absorption values and voids index for the investigated concrete compositions. These results are important because they contribute to the study of the pozzolanic activity of the waste, since this type of results also helps in the effect of filling the concrete. In fact, pozzolans have a chemical effect, obtained through the pozzolanic reaction, in which the material contributes to the strength of the concrete, although there was a small loss of properties. However, pozzolans also have a physical effect of filling voids, which was verified by the density results in Figure 5, where there was a statistical equivalence in the values obtained. If the waste did not act filling the voids there would be a change in the density of the material [57].

The density results in Figure 5 is confirmed by the verified values of capillarity in the concrete, illustrated in Figure 6. By the capillarity curve, it is verified that the compositions of 0 and 10% present the same pattern, while the composition of 20% presents less absorption than the other compositions in the initial hours, up to 50 h, presenting greater capillarity after this period. However, the evaluated values were not discrepant, with practically the same behavior among the studied concretes, which confirms the density values obtained.

It is also verified by the water absorption values and voids index, Figure 7, that there was a tendency to increase the average values obtained as the percentage of waste increased. However, this increase was not statistically effective, because there was an equivalence between the intervals evaluated due to the standard deviation associated with the test results. Thus, the most accurate discussion of the results was that they were statistically equivalent, which led to the conclusion that the CCW acts as a filling effect, since the water absorption, by immersion and capillarity and density and index of empty, remained equivalent to the reference composition.

**Figure 8:**
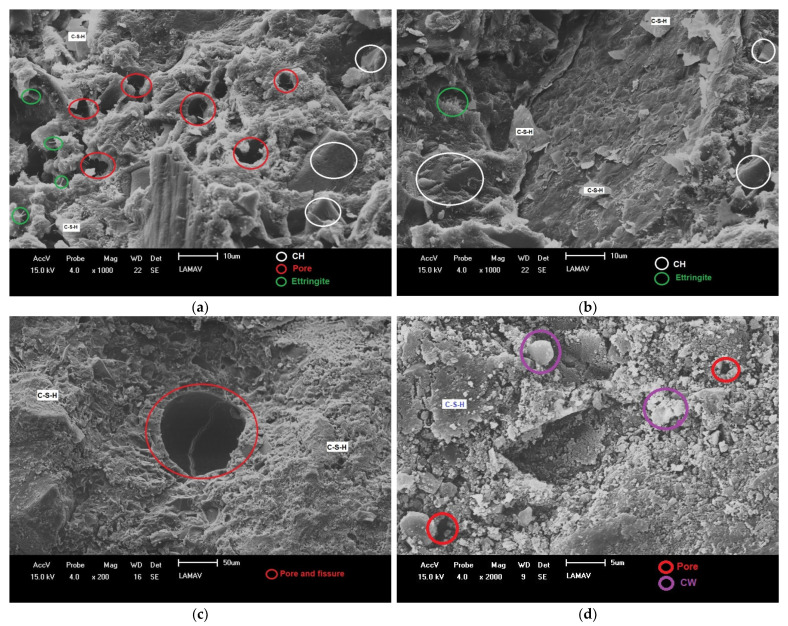
SEM of the studied concretes: (**a**) and (**b**) C0%; (**c**) and (**d**) C20%.

Figure 8 shows SEM fractographic results of the 0 and 20% compositions. In Figure 8a,b, corresponding to the reference concrete, the presence of C–S–H, ettringite and CH crystals was verified. It is also possible to identify capillary voids in the fracture surface of the C0% concrete. In Figure 8c,d, the presence of CH was not verified, which is an indication that this C20% concrete had the CH quantity reduced in the concrete composition. This might be attributed to the efficiency of the pozzolanic reaction. The amount of ettringite also showed a reduction. It is also possible to detect the presence of pores and cracks in the C20% concrete, in addition to lose particles of ceramic waste, which did not react and were present in the isolated form within the material. This may be an indication that when using 20% of the CCW, there was not enough CH to process the material’s reaction, which explains the reduction in compressive strength for the C20% concrete. That is, using 10% of CCW, there was a pozzolanic reaction in amount sufficient to maintain the strength parameters. However, using 20 wt.% of CCW, there was not enough CH to process the pozzolanic reaction. This is proven by the absence of CH in the C20% concrete and explains the presence of isolated particles of CCW, observed by SEM in Figure 8d. These also explain the reduction in strength that occurred for the 20% CCW composition, which reduced from 34.83 MPa for the reference composition at 28 days to 27.82 MPa, a percentage reduction of 20.12%. This reduction can be attributed to the depletion of the available portlandite for the occurrence of the pozzolanic reaction observed in Figure 8d.

## 4. Suggestions for Future Work

The results obtained in this research can be increased with the realization of future works presenting continuity of the theme. It is suggested:

- Evaluation of the use of other percentages of CCW in the properties of the studied concrete. For example, using 5 or 15% of the waste as a substitute for cement;
- Use of the plasticizer additive to reduce the water/cement (w/c) ratio, making it possible to obtain a concrete with superior strength, aiming to study how the CCW affects the properties of concrete with a lower w/c ratio;
- Evaluation of the compactness of concrete using ceramic residue using techniques of porosometry, electrical resistivity or ultrasound;
- Evaluation of the rheological properties of concrete containing CCW;
- Evaluation of the durability of concretes containing CCW through high temperature tests, salt spray, ice–thaw cycles, immersion and drying tests and/or chloride attack;
- Evaluation of the durability of concretes containing CCW in a situation of water and gas permeability;
- Life cycle analysis of concretes containing CCW comparing them to concretes without incorporating waste;
- Economic analysis, using the principles of circular economy, of the application of CCW as a substitute for cement in concrete.

## 5. Conclusions

It can be concluded that the CCW presented a variation in electrical conductivity of 1.21 mS/cm, which made possible its classification in high pozzolanicity, corroborating with the chemical and mineralogical characterization, which showed the presence of metakaolinite with amorphous regions in CCW. Through the index of pozzolanic activity and the characteristic compression strength, we observed that the concrete even with 20% of CCW presents potential structural applications in urban environments, where the pollution aggressiveness was intermediate and concrete with compression strength higher than 25 MPa was required. It is observed, for example, that the compressive strength for the C10 composition was 27.2 MPa at 14 days and 31.42 MPa at 28 days, while the C20 composition showed compressive strength of 24.5 MPa at 14 days and 27.82 MPa at 28 days. The reduction in compressive strength, comparing the compositions with the reference, was attributed to the exhaustion of portlandite in the hydration of the cement, due to the pozzolanic reaction. However, even with the reduction, the observed values were compatible with concretes with structural applications.

In addition to the chemical and pozzolanic effect, illustrated by the result of resistance to compression, it was observed that the ceramic residue had an effect on the physical properties. This was verified through the results of density, capillarity, water absorption and voids index. From a physical point of view, it is observed that the CCW had a physical filling effect, because even using smaller amounts of cement, the ceramic residue maintained the density and voids of the concretes with statistical equivalence.

In the SEM microstructure analysis, it was confirmed that the CCW had a significant effect as a pozzolanic constituent, since the amount of portlandite detected in the reference composition, 0% CCW, was substantially reduced in the composition with 20% CCW (optimum mixture), proving the effectiveness of the pozzolanic activity. Finally, it can be concluded that the CCW has a pozzolanic potential and is feasible to be used as a partial substitute for OPC in 20% for the production of structural concrete.

## Figures and Tables

**Table 1:** Clay ceramic waste applied as pozzolan.

Authors	Waste Characteristics	Properties	Pozzolanicity Test	Percentage Replaced	Conclusions about Mechanical Strength
Kannan et al. (2017) [34]	Waste extracted from polishing ceramic glazes containing silica and alumina. (69.4% SiO_2_; 18.2% Al_2_O_3_; 3.53% MgO; 3.19% Na_2_O)	Compressive strength, permeability, X-ray Diffraction (XRD) and Magic Angle Spinning Nuclear Magnetic Resonance (MAS-NMR).	Frattini test	0, 10, 20, 30, 40%	The compressive strength dropped from 63.8 (0%) to 57.5 (10%), 52.3 (20%), 49 (30%) and 42.6 MPa (40%), concluding that the percentages of 30% and 40% of the waste are harmful to strength.
Irassar et al. (2014) [35]	Waste extracted from an Argentine ceramic containing quartz, feldspar, anortite and hematite. (51.3% SiO_2_; 20% Al_2_O_3_; 11.5% CaO; 6% Fe_2_O_3_; 3.2% K_2_O)	Calorimetry and XRD.	Frattini test	8, 16, 24, 32, 40%	They did not evaluate the mechanical strength, only the kinetics of the reactions.
Pokorný et al. (2014) [36]	Waste extracted from a Spanish ceramic block industry containing gypsum, quartz, microclimate, albite, muscovite and smaller amounts of hematite (37% SiO_2_; 14.28% Al_2_O_3_; 11.14% CaO; 4.77% Fe_2_O_3_; 17.9% SO_3_)	Compressive strength, porosity, density, thermal conductivity.	Unrealized	0, 8, 16, 24, 32%	The compressive strength values were: 59.4 (0%), 45.7 (10%), 43.9 (16%), 26.6 (24%) and 21.7 MPa (32%). There was a high reduction in strength to 24% and 32% due to the presence of gypsum, eliminating the possibility of using these percentages.
Vejmelková et al. (2014) [37]	Ground brick waste from the production of thermal insulating blocks (54.97% SiO_2_; 14.3% Al_2_O_3_; 15.2% CaO; 4.8% Fe_2_O_3_)	Compressive strength, porosity, density, thermal conductivity.	Unrealized	0, 10, 20, 40, 60%	The compressive strength values were: 43 (0%), 41.2 (10%), 40.1 (20%), 26.9 (40%) and 24.5 MPa (60%). The percentages of 40% and 60% were discarded due to the high drop in strength.
Cheng et al. (2014) [38]	Ceramic polishing waste extracted from a Chinese industry composed of quartz and mullite (69.02% SiO_2_; 16.04% Al_2_O_3_; 4.08% MgO; 3.15% Na_2_O)	Compressive strength and durability tests (acid attack), XRD and Scanning Electron Microscope (SEM).	Unrealized	0, 10, 20, 30, 40%	The strength dropped from 45.5 to 44.68 (10%), 41.35 (20%), 33.19 (30%) and 32.97 (40%). An excessive drop is observed for the compositions of 30% and 40%.
Cheng et al. (2016) [39]	Ceramic polishing waste extracted from a Chinese industry composed of quartz and mullite (69.02% SiO_2_; 16.04% Al_2_O_3_; 4.08% MgO; 3.15% Na_2_O)	Compressive strength and chloride permeability tests, XRD, SEM and Energy-dispersive X-ray Spectroscopy (EDS).	Unrealized	0, 10, 20, 30, 40%	The strength dropped from 45.5 to 44.68 (10%), 41.35 (20%), 33.19 (30%) and 32.97 (40%). An excessive drop is observed for the compositions of 30% and 40%.
Pacheco-Torgal and Jalali (2011) [40]	Ceramic waste from burnt bricks composed of quartz and hematite, calcite, cristobaltite and feldspar in smaller quantities. (51.7% SiO_2_; 18.2% Al_2_O_3_; 6.1% Fe_2_O_3_; 6.1% CaO)	Compressive strength, porosity, water absorption, oxygen and water permeability, durability in attack by chlorides.	Unrealized	0 and 20%	The strength results obtained for concrete with 20% were excellent (39.7 MPa) when compared to the reference composition (40.4 MPa).
Reiterman et al. (2014) [41]	Ceramic waste is not characterized.	Compressive strength, density, acid attack durability.	Unrealized	0, 5, 10, 15, 20, 25, 30%.	Strength values: 61 (0%); 64.3 (5%); 62.9 (10%); 59.5 (15%), 58.8 (20%), 51.8 (25%) and 46.2 (30%). A considerable drop in strength is observed to 25% and 30%.
Wang (2009) [42]	Ceramic waste is not characterized.	Compressive strength, linear shrinkage, heat of hydration, porosity, SEM.	Unrealized	0, 10, 20, 50%.	Strength values: 55.6 (0%), 49.8 (10%), 46.5 (20%) and 32.5 (50%). There is a high reduction in strength for the composition of 50%.

**Table 2:** Chemical composition (%) of the waste (CCW).

SiO_2_	A_2_O_3_	Fe_2_O_3_	K_2_O	Na_2_O	TiO_2_	P_2_O_5_	CaO
56.80	32.30	3.70	1.60	0.70	1.10	0.25	0.98

**Table 3:** Proportion of materials used in concrete.

Concrete	Portland Cement	Fine Aggregate	Coarse Aggregate	Clay Ceramic Waste	Water/Cement	Slump Teste (cm)
C0%	1.00	1.38	2.95	0.00	0.49	70 ± 10
C10%	0.90	1.38	2.95	0.10	0.54	
C20%	0.80	1.38	2.95	0.20	0.61	

**Table 4:** Pozzolanic activity by Luxán (mS/cm).

Information	Result
Initial conductivity	7.18
Final conductivity	8.39
Conductivity variation	1.21
Classification	High pozzolanicity

## Data Availability

Data sharing is not applicable to this article.
